# miR-139-5p sponged by LncRNA NEAT1 regulates liver fibrosis via targeting β-catenin/SOX9/TGF-β1 pathway

**DOI:** 10.1038/s41420-021-00632-8

**Published:** 2021-09-16

**Authors:** Qi Wang, Song Wei, Lei Li, Qingfa Bu, Haoming Zhou, Wantong Su, Zheng Liu, Mingming Wang, Ling Lu

**Affiliations:** 1 School of Medicine, Southeast University, Nanjing, China; 2 Jiangsu Cancer Hospital, Jiangsu Institute of Cancer Research, Nanjing Medical University Affiliated Cancer Hospital, The First Affiliated Hospital of Nanjing Medical University, Research Unit of Liver Transplantation and Transplant Immunology, Chinese Academy of Medical Sciences, Key Laboratory of Liver Transplantation, Chinese Academy of Medical Sciences, NHC Key Laboratory of Living Donor Liver Transplantation, Nanjing, China; 3 Jiangsu Key Lab of Cancer Biomarkers, Prevention and Treatment, Collaborative Innovation Center for Personalized Cancer Medicine, Nanjing Medical University, Nanjing, China; 4 State Key Laboratory of Reproductive Medicine, Nanjing, China

**Keywords:** Cell biology, Diseases

## Abstract

Liver fibrosis is a patho-physiological process which can develop into cirrhosis, and hepatic carcinoma without intervention. Our study extensively investigated the mechanisms of lncRNA NEAT1 and miR-139-5p in regulating liver fibrosis progression. Our results demonstrated that the expression of lncRNA NEAT1 was increased and the expression of miR-139-5p was decreased in fibrotic liver tissues. LncRNA NEAT1 could sponge miR-139-5p and promoted hepatic stellate cells (HSCs) activation by directly inhibiting the expression of miR-139-5p. The co-localization of lncRNA NEAT1 with miR-139-5p was shown in the cytosols of activated HSCs. miR-139-5p upregulation could suppress the expression of β-catenin. The overexpression of β-catenin promoted HSCs activation. Moreover, we found that β-catenin could interact with SOX9 promoted HSCs activation. Our further studies demonstrated that SOX9 could bind with the TGF-β1 promoter and promoted the transcription activity of TGF-β1. The upregulation of TGF-β1 further promoted HSCs activation. In vivo study also suggested that lncRNA NEAT1 knockdown and miR-139-5p overexpression alleviated murine liver fibrosis. LncRNA NEAT1 exacerbated liver fibrosis by suppressing the expression of miR-139-5p. Collectively, our study suggested that miR-139-5p sponged by lncRNA NEAT1 regulated liver fibrosis via targeting β-catenin/SOX9/TGF-β1 Pathway.

## Introduction

Liver fibrosis is a chronic disease that a repairing procedure happens upon liver injury, resulting in excessive generation of extracellular matrix (ECM), mainly containing type I and III collagens, by the activated HSCs (aHSCs) [1]. Hepatic fibrosis progressively restricts normal liver regeneration, thus increasing the risk of liver failure. Hepatic fibrosis also generates a permissive micro-environment for the development of liver cancer through mechanisms that are not fully elucidated [2]. Liver fibrosis is associated with persistent stimulation with fibrogenic mediators and characterized by the upregulated expression of α-smooth muscle actin and other myofibroblast intracellular microfilaments. Activated HSCs migrate to the site of injury and secrete ECM to produce a fibrous scar [3, 4]. Since the HSCs activation play an essential role in fibrogenesis, targeting aHSCs is an attractive strategy for treating liver fibrosis [5].

In recent years, long non-coding RNAs (lncRNAs) and microRNAs (miRNAs) have become the star molecules in the study of many diseases. lncRNAs mainly act as sponges of target miRNAs, which are non-coding transcripts with a length of over 200 nucleotides [6] participating in modulating diverse processes in cells, including gene expression and various biological functions [7]. MicroRNAs (miRNAs) are single-stranded, small (22- to 25-nt), non-coding RNA molecules which can regulate cell proliferation and survival by binding to complementary target mRNAs, resulting in the inhibition of mRNA translation or degradation [8, 9]. LncRNAs and miRNAs are closely related to fibrosis disease in lung, liver, kidney and myocardium and they are considered to be new therapeutic targets in fibrosis because of functional regulation in the fibrosis pathology. For example, LncRNA NEAT1 promotes pulmonary fibrosis through miR-9-5p/TGF-β signaling pathway [10]. LncRNA NEAT1 accelerates the renal fibrosis caused by diabetic nephropathy through AKT/mTOR signaling pathway [11]. LncRNA NEAT1-enriched vesicles secreted by hypoxic cardiomyocytes excerabate cardiac fibrosis [12]. Importantly, lncRNA NEAT1 was positively correlated with the expressions of α-SMA and Col1A1 in human cirrhosis [13]. Therefore, lncRNA NEAT1 may be a potential biomarker for liver fibrosis and further studies are needed to explore the specific mechanism of NEAT1 in the pathogenesis of liver fibrosis. Moreover, miRNAs also exerted important regulatory effects in liver diseases. miR-139-5p suppresses hepatocellular carcinoma cell growth by downregulating karyopherin alpha 2 [14]. High quantity and variety of microRNA-139-5p isoforms exert suppressive role in hepatocellular carcinoma [15]. NEAT1 induces hepatocellular carcinoma progression via sponging hsa-miR-139-5p and upregulating TGF-β1 [16]. A recent study indicates that miR-139-5p inhibits epithelial-mesenchymal transition and fibrosis in post-menopausal women with interstitial cystitis by targeting LPAR4 via the PI3K/Akt signaling pathway [17]. Another study demonstrated that miR-139-5p could regulate inflammatory and contribute to the progression of primary biliary cholangitis by suppressing the expression of c-FOS [18]. However, the role of miR-139-5p in liver fibrosis remains unreported. Bioinformatic analysis predicted that miR-139-5p was a potential target of the lncRNA NEAT1, which might promote liver fibrosis essentially.

β-catenin acts as a transcription factor promoting the expression of T cell factor-lymphoid enhancer factor-dependent gene upon translocating into the nucleus [19]. Studies had shown that β-catenin performed an important role in fibrotic diseases. A study suggested that the expressions of Ogn and β-catenin were high in mice with myocarditis [20]. Another study demonstrated that cannabinoid receptor type 2 promoted kidney fibrosis by modulating β-catenin signaling [21]. Icaritin represses skin fibrosis via regulating AMPK and Wnt/β-catenin signaling [22]. HSC-specific inhibition of β-catenin may have therapeutic benefits for cirrhotic portal hypertension [23]. However, the mechanisms that increased expression of β-catenin in liver fibrosis remain poorly known. Bioinformatic analysis indicated that β-catenin could be directly targeted by miR-139-5p, which has not been experimentally validated so far in liver fibrosis. Moreover, the pathogenic role of increased expression of β-catenin in fibrogenesis has been mediated by its promotion of SOX9 expression [24]. In epidermal morphogenesis, β-catenin activation greatly upregulated the expression of SOX9 [25]. In pulmonary branching morphogenesis, β-catenin inhibits the expression of SOX9 in basal cells critically [26], and SOX9 could enhance hepatic ischemia/reperfusion injury by promoting TGF-β1 expression [27]. However, the association between SOX9 and β-catenin in liver fibrosis still remains largely unknown.

Herein, our study verified that lncRNA NEAT1 could target and suppress the expression of miR-139-5p directly to promote HSCs activation and excerabate liver fibrosis via β-catenin/SOX9/TGF-β1 axis. Our study revealed a new signaling pathway underlying liver fibrosis, laid a foundation for the early diagnosis of non-coding-RNA-based liver fibrosis and the development of novel anti-fibrosis drugs.

## Results

### LncRNA NEAT1 is upregulated and miR-139-5p is downregulated in fibrotic liver tissues and activated HSCs

**Fig. 1:**
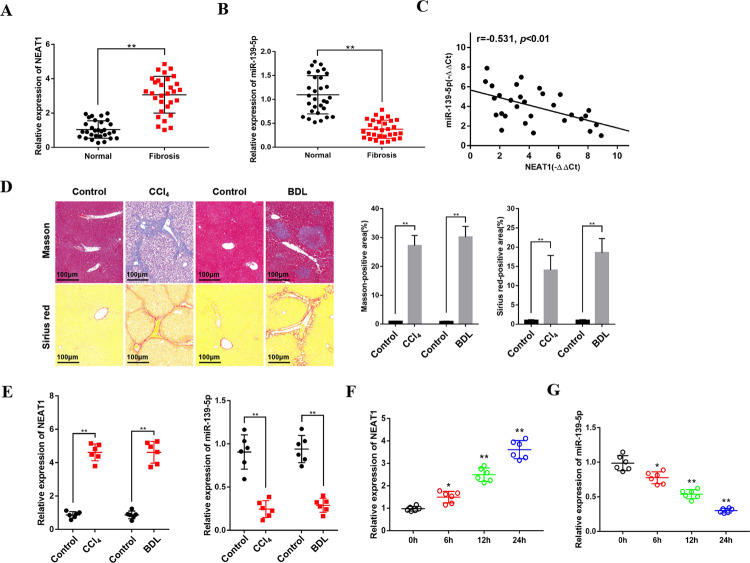
Expression of NEAT1 is upregulated and miR-139-5p is downregulated in liver fibrosis. **A** The expression of NEAT1 was upregulated in human liver fibrosis tissues. *n* = 30/group. **B** The expression level of miR-139-5p was downregulated in human liver fibrosis tissues. *n* = 30/group. **C** The correlation between NEAT1 and miR-139-5p expression levels in fibrotic liver tissues from patients was evaluated with Pearson’s correlation analysis, *n* = 30. **D** The liver tissues from mice treated with CCl_4_ for 8 weeks and BDL for 2 weeks were performed with Masson staining and Sirius red staining. *n* = 6/group, original magnification×200. **E** The expression level of NEAT1 was upregulated and the expression level of miR-139-5p was downregulated in murine liver fibrosis tissues. *n* = 6/group. **F** The expression level of NEAT1 was upregulated in activated LX-2 cells stimulated with 10 ng/mL TGF-β1 in a time-dependent manner. *n* = 6/group. **G** The expression level of miR-139-5p was decreased in activated LX-2 cells stimulated with 10 ng/mL TGF-β1 in a time-dependent manner. *n* = 6/group. Graph represents mean ± SD. *P < 0.05, **P < 0.01.

To investigate the relationship between lncRNA NEAT1 and miR-139-5p in liver fibrosis, qRT-PCR was performed to examine the expression of lncRNA NEAT1 and miR-139-5p in fibrotic liver tissues and HSC line LX-2 cells. The results of qRT-PCR demonstrated that NEAT1 was significantly increased in human fibrotic liver tissues (Fig. 1A). The expression of miR-139-5p was significantly decreased in human fibrotic liver tissues (Fig. 1B). Meanwhile, the negative correlation between NEAT1 levels and miR-139-5p expression was observed in patient fibrotic tissues (Fig. 1C). Moreover, mice were subjected to CCl_4_ or BDL to develop liver fibrotic models (Fig. 1D). The results indicated that the expression of NEAT1 was greatly upregulated and the expression of miR-139-5p was greatly downregulated in murine fibrotic liver tissues (Fig. 1E). Then, we also determined the expression of NEAT1 in activated LX-2 cells stimulated with 10 ng/mL TGF-β1 at different times, which showed a time-dependent increase (Fig. 1F), and the expression of miR-139-5p in activated LX-2 cells stimulated with TGF-β1, which showed a time-dependent decrease (Fig. 1G). These results reveal that NEAT1 upregulation and miR-139-5p downregulation might be closely correlated with liver fibrogenesis.

### LncRNA NEAT1 targets and suppresses miR-139-5p expression in activated HSCs

**Fig. 2:**
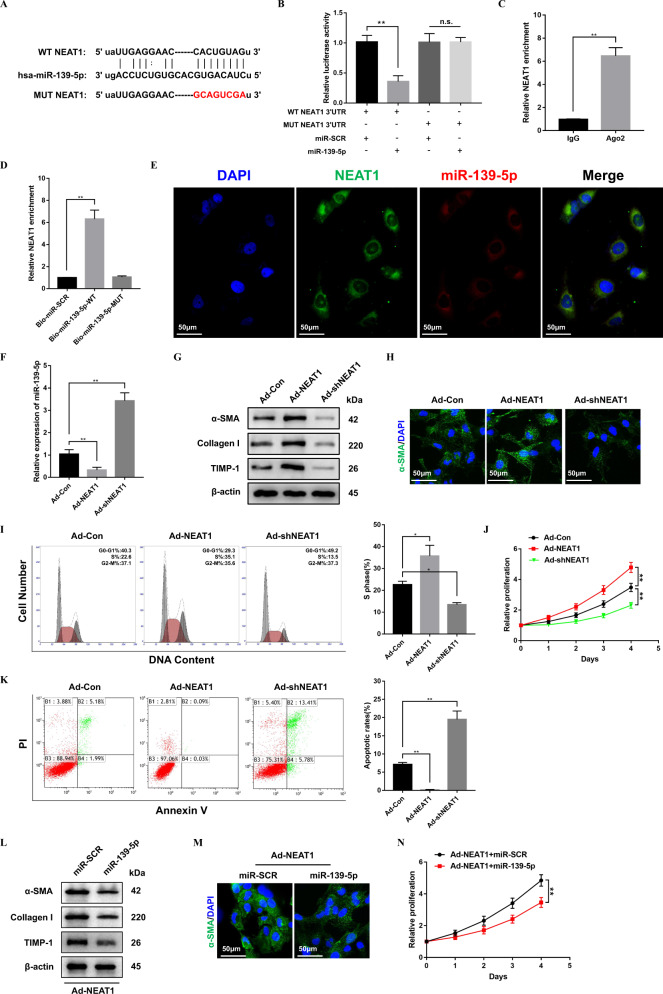
miR-139-5p is a target of NEAT1. **A** A schematic diagram demonstrated the putative sites of miR-139-5p binding with NEAT1. **B** Relative luciferase activities of pmirGLO-NEAT1-WT or pmirGLO-NEAT1-MUT were detected in HEK293T cells. **C** RIP experiments were carried out with Ago2 antibody on extracts from LX-2 cells. Relative expression level of NEAT1 was shown as fold enrichment in Ago2 relative to IgG immunoprecipitates by qRT-PCR. **D** Interaction between NEAT1 and miR-139-5p was verified by pull-down assay. *n* = 3/group. **E** RNA in situ hybridization was performed to observe the co-localization between miR-139-5p and NEAT1. *n* = 3/group. Next, LX-2 cells were transfected with Ad-NEAT1 or Ad-shNEAT1 for 48 h. **F** qRT-PCR was performed to detect the expression level of miR-139-5p in LX-2 cells transfected with Ad-NEAT1 or Ad-shNEAT1. *n* = 3/group. **G** The protein levels of α-SMA, Collagen-I, and TIMP-1 in LX-2 cells transfected with Ad-NEAT1 or Ad-shNEAT1 were detected by western blotting. Representative of three experiments. **H** Confocal laser microscopy was used to analyze the immunofluorescence staining for α-SMA (green) in LX-2 cells after transfection with Ad-NEAT1 or Ad-shNEAT1. DAPI was used to stain nuclei, blue. Original magnification×400. Representative of three experiments. **I** Flow cytometry was performed to analyze the cell-cycle distribution of LX-2 cells transfected with Ad-NEAT1 or Ad-shNEAT1. Representative of three experiments. **J** CCK8 assay was performed to detect the proliferation of LX-2 cells transfected with Ad-NEAT1 or Ad-shNEAT1. **K** Flow cytometry was performed to detect the cell apoptosis of LX-2 cells transfected with Ad-NEAT1 or Ad-shNEAT1. Representative of three experiments. **L** The protein levels of α-SMA, Collagen-I, and TIMP-1 in LX-2 cells transfected with Ad-NEAT1 and miR-139-5p were detected by western blotting. Representative of three experiments. **M** Confocal laser microscopy was used to analyze the immunofluorescence staining for α-SMA (green) in LX-2 cells after transfection with Ad-NEAT1 and miR-139-5p. DAPI was used to stain nuclei, blue. Original magnification×400. Representative of three experiments. **N** CCK8 assay was performed to detect the proliferation of LX-2 cells transfected with Ad-NEAT1 and miR-139-5p. Graph represents mean ± SD. *P < 0.05, **P < 0.01.

The correlation between lncRNAs and microRNAs in liver fibrosis remain largely unknown. Here, ChipBase, LncRNAdb, and StarBase were used in our study to determine the association between NEAT1 and miR-139-5p. Then, we performed luciferase reporter assay to verify that miR-139-5p was a target of NEAT1 (Fig. 2A). LX-2 cells were transfected with miR-139-5p mimics and the luciferase reporter plasmids, which contained the wild type of NEAT1 or the mutant type of NEAT1. The result showed a decreased reporter activity compared to the negative controls (Fig. 2B). Then, RIP assay was performed to test whether miR-139-5p could be sponged by NEAT1. The experiment demonstrated that NEAT1 and miR-139-5p were more abundant in Ago2 pellet than in IgG pellet (Fig. 2C). Moreover, RNA pull-down assay with biotinylated miR-139-5p (Bio-miR-139-5p) probe increased the level of NEAT1 compare to the Bio-miR-SCR or Bio-miR-139-5p-Mut group (Fig. 2D). Notably, the experiments of fluorescence in situ hybridization (FISH) suggested a co-localization between miR-139-5p and NEAT1 in the cytoplasm of activated LX-2 cells (Fig. 2E). Next, to further investigate the role of NEAT1 in fibrogenesis, we elevated and suppressed the expression of NEAT1 in activated HSCs transfected with Ad-NEAT1 or Ad-shNEAT1. The results of qRT-PCR indicated that overexpression of NEAT1 decreased the expression of miR-139-5p and downregulation of NEAT1 increased the expression of miR-139-5p in HSCs (Fig. 2F). NEAT1 overexpression in LX-2 cells increased the protein levels of profibrotic markers, including α-SMA, Collagen-I, and TIMP-1 and the downregulation of NEAT1 in LX-2 cells decreased the protein levels of profibrotic markers (Fig. 2G). Likewise, the analysis of immunofluorescence demonstrated the same change of α-SMA in LX-2 cells treated with Ad-NEAT1 or Ad-shNEAT1 (Fig. 2H). In addition, overexpression of NEAT1 also significantly increased the proportion of S phase cells and promoted the cell proliferation, downregulation of NEAT1 in LX-2 cells decreased the proportion of S phase cells and inhibited the cell proliferation (Fig. 2I, J). Moreover, NEAT1 overexpression also resulted in decreased apoptosis in LX-2 cells, downregulation of NEAT1 resulted in increased apoptosis in LX-2 cells (Fig. 2K). Meanwhile, inhibited cell activation (Fig. 2L, M) and proliferation (Fig. 2N) under miR-139-5p overexpression in Ad-NEAT1 transfected HSCs further validated that NEAT1 enhanced HSCs activation by suppressing miR-139-5p expression. These results demonstrate that miR-139-5p can act as a target of NEAT1 to modulate HSCs activation.

### β-catenin is overexpressed in fibrotic liver tissues and miR-139-5p upregulation suppresses β-catenin expression

**Fig. 3:**
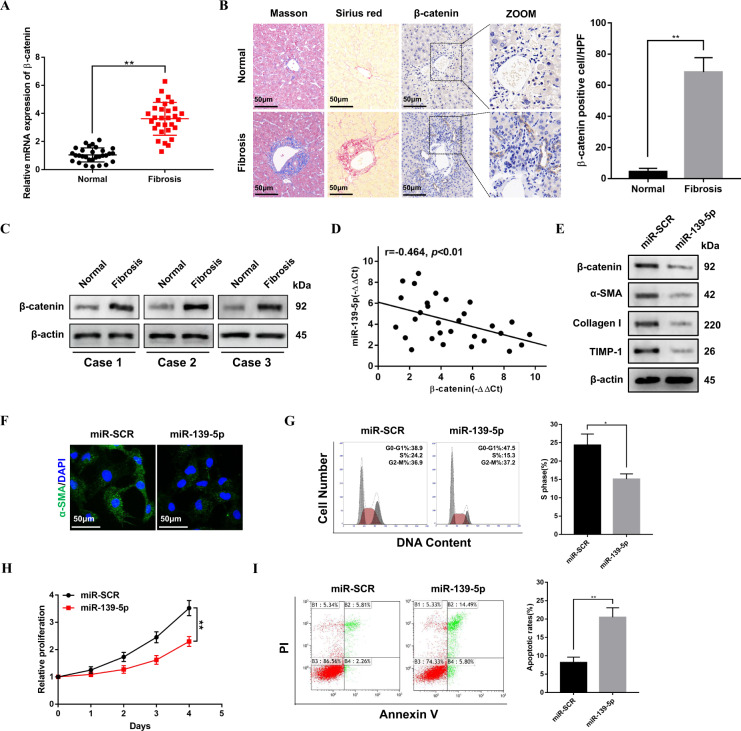
β-catenin is overexpressed in fibrotic liver tissues and miR-139-5p upregulation suppresses β-catenin expression. **A** The mRNA expression level of β-catenin was upregulated in human fibrotic liver tissues. *n* = 30/group. **B** The sections of human liver tissues were treated with Masson staining, Sirius red staining and Immunohistochemical staining of β-catenin. *n* = 6/group, original magnification×200. **C** The protein levels of β-catenin in normal or fibrotic liver tissues from patients were examined by western blotting. Representative of three experiments. **D** The correlation between the expression levels of β-catenin and miR-139-5p in patient fibrotic liver tissues was evaluated with Pearson’s correlation analysis, *n* = 30. **E** The protein levels of β-catenin, α-SMA, Collagen-I, and TIMP-1 in LX-2 cells infected with miR-139-5p mimics were detected by western blotting. Representative of three experiments. **F** Immunofluorescence staining for α-SMA (green) in LX-2 cells transfected with miR-139-5p mimics was analyzed by confocal laser microscopy. DAPI was used to stain nuclei, blue. Original magnification×400, Representative of three experiments. **G** The cell-cycle distribution of LX-2 cells infected with miR-139-5p mimics was analyzed by flow cytometry. Representative of three experiments. **H** Proliferation of LX-2 cells infected with miR-139-5p mimics was analyzed by CCK8 assay. **I** The cell apoptosis of LX-2 cells infected with miR-139-5p mimics was analyzed by flow cytometry. Representative of three experiments. Graph represents mean ± SD. *P < 0.05, **P < 0.01.

A previous study has indicated that β-catenin could promote HSCs activation and excerabate liver fibrosis [23]. However, whether miR-139-5p could modulate β-catenin expression in HSCs activation and fibrotic liver remains undetermined. The results of qRT-PCR, immunohistochemistry and western blotting indicated that β-catenin is upregulated in patient fibrotic liver tissues (Fig. 3A–C). In addition, the negative correlation between the levels of miR-139-5p and the expression of β-catenin was detected in patient fibrotic liver tissues (Fig. 3D). Next, HSCs were transfected with miR-139-5p mimics to overexpress miR-139-5p. As shown in Fig. 3E, miR-139-5p overexpression inhibited the protein levels of β-catenin and profibrotic markers, including α-SMA, Collagen-I, and TIMP-1, further verified by decreased α-SMA immunofluorescence staining (Fig. 3F). Meanwhile, miR-139-5p overexpression significantly decreased the proportion of S phase cells and inhibited the cell proliferation (Fig. 3G, H). Moreover, miR-139-5p overexpression also resulted in increased apoptosis in LX-2 cells (Fig. 3I). Our findings suggest that β-catenin is upregulated in fibrotic liver tissues and miR-139-5p overexpression suppresses β-catenin expression.

### miR-139-5p regulates β-catenin expression by binding to the 3’ UTR of its mRNA directly

**Fig. 4:**
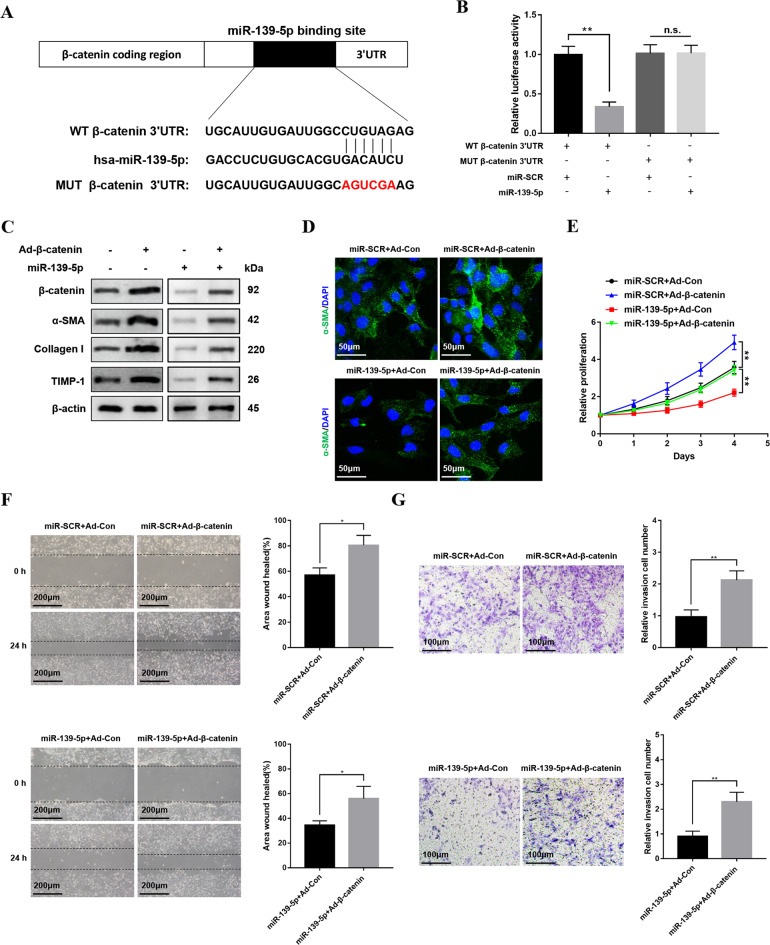
miR-139-5p regulates the expression of β-catenin by binding to the 3’ UTR of its mRNA directly. **A** schematic diagram suggested the putative binding sites of miR-139-5p with respect to β-catenin. **B** WT HLF 3’UTR or MUT HLF 3’UTR luciferase reporter activity in LX-2 cells was detected by dual-luciferase reporter assay. **C** The protein levels of β-catenin, α-SMA, Collagen-I, and TIMP-1 in LX-2-pre-miR-139-5p cells infected with Ad-β-catenin were detected by western blotting. Representative of three experiments. **D** Immunofluorescence staining for α-SMA (green) in LX-2-pre-miR-139-5p cells after transfection with Ad-β-catenin was observed by confocal laser microscopy. DAPI was used to stain nuclei, blue. Original magnification×400, Representative of three experiments. **E** Proliferation of LX-2-pre-miR-139-5p cells infected with Ad-β-catenin was analyzed by CCK8 assay. **F** The wound-healing assay of LX-2-pre-miR-139-5p cells infected with Ad-β-catenin or Ad-Con was performed to analyze the migration capability. Representative of three experiments. **G** The transwell assay of the LX-2-pre-miR-139-5p cells transfected with Ad-β-catenin or Ad-Con was carried out to observe the migration capability, representative of three experiments. The number of cells was calculated from different fields. Graph represents mean ± SD. *P < 0.05, **P < 0.01.

To further explore how miR-139-5p regulates β-catenin and affects HSCs activation, bioinformatics softwares including TargetScan, miRanda and miRbase were used in our study. Our study predicted that miR-139-5p could bind with the 3’-UTR of β-catenin (Fig. 4A). Luciferase reporter gene assay further indicated that miR-139-5p overexpression repressed the luciferase activity of wild-type β-catenin 3’-UTR (WT β-catenin 3’-UTR), but not with mutant β-catenin 3’-UTR (MUT β-catenin 3’-UTR) (Fig. 4B). To further verify that miR-139-5p modulated HSC activation and proliferation by targeting β-catenin, β-catenin was overexpressed in the cultured LX-2 cells transfected with miR-139-5p mimics using adenovirus. the overexpression of β-catenin promoted the expression of profibrotic markers including α-SMA, Collagen-I, and TIMP-1 (Fig. 4C), further verified by increased immunofluorescence staining of α-SMA (Fig. 4D). Meanwhile, the overexpression of β-catenin promoted the cells proliferation (Fig. 4E). In addition, the overexpression of β-catenin significantly promoted the cells migration (Fig. 4F, G). These results suggest that miR-139-5p regulates HSCs activation by binding to the 3’UTR of β-catenin mRNA directly.

### miR-139-5p regulates HSCs activation depending on the interaction between β-catenin and SOX9

**Fig. 5:**
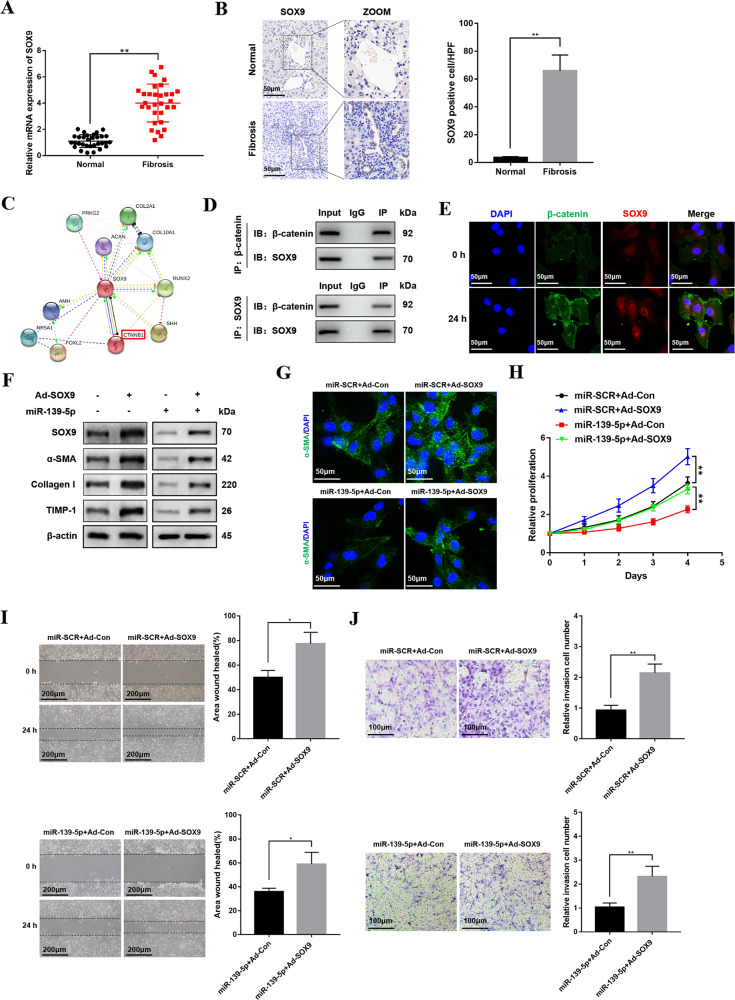
miR-139-5p regulates HSCs activation depending on the interaction between β-catenin and SOX9. **A** The mRNA expression level of SOX9 was upregulated in human fibrotic liver tissues. *n* = 30/group. **B** The sections of human liver tissues were treated with SOX9 immunohistochemical staining, *n* = 6/group, original magnification×200. **C** Predicted protein network visualization with STRING. The network nodes represented proteins. These proteins were clustered with k-means clustering algorithms. **D** Co-immunoprecipitation assays revealed that β-catenin could bind with SOX9 in activated LX-2 cells. **E** Moreover, Dual immunofluorescence staining of activated LX-2 cells using anti-β-catenin and anti-SOX9 antibodies was performed. Representative of three experiments. DAPI was used to stain the nuclei, original magnification×400. **F** The protein levels of SOX9, α-SMA, Collagen-I, and TIMP-1 in LX-2-pre-miR-139-5p cells infected with Ad-SOX9 were detected by western blotting. Representative of three experiments. **G** Immunofluorescence staining for α-SMA (green) in LX-2-pre-miR-139-5p cells infected with Ad-SOX9 was analyzed by confocal laser microscopy. DAPI was used to stain the nuclei, blue. Original magnification×400, Representative of three experiments. **H** Proliferation of LX-2-pre-miR-139-5p cells infected with Ad-SOX9 was analyzed by CCK8 assay. **I** The wound-healing assay of LX-2-pre-miR-139-5p cells infected with Ad-SOX9 or Ad-Con was performed to observe the migration capability. Representative of three experiments. **J** The transwell assay of the LX-2-pre-miR-139-5p cells infected with Ad-Ad-SOX9 or Ad-Con was performed to analyze the migration capability, representative of three experiments. The number of cells was calculated from different fields. Graph represents mean ± SD. *P < 0.05, **P < 0.01.

A study has indicated that SOX9 could promote HSCs activation and exacerbate liver fibrosis [28]. Nevertheless, the mechanism how SOX9 regulates HSCs activation and fibrotic liver remains unclear. The results of qRT-PCR and immunohistochemistry demonstrated that SOX9 was upregulated in patient fibrotic liver tissue (Fig. 5A, B). To explore the functional interactions between SOX9 with other proteins expression in LX-2 cells, the STRING (https://string-db.org/cgi/input.pl) database was used to predict protein interaction network. As shown in Fig. 5C, there are ten proteins correlating with SOX9 closely. β-catenin translated by CTNNB1 was the most related protein interacting with SOX9. Meanwhile, coimmunoprecipitation assays indicated that β-catenin could bind with SOX9 in activated LX-2 cells (Fig. 5D). Moreover, dual immunofluorescent staining also revealed increased β-catenin and SOX9 co-expression in activated LX-2 cells (Fig. 5E). To further elucidate the important role of SOX9 in activated LX-2 cells, we overexpressed SOX9 in activated LX-2 cells after transfection with miR-139-5p mimics and found that SOX9 overexpression promoted profibrotic markers expression including α-SMA, Collagen-I, and TIMP-1 (Fig. 5F), further verified by increased α-SMA immunofluorescence staining (Fig. 5G). Meanwhile, the overexpression of SOX9 could promote the cell proliferation (Fig. 5H). In addition, the overexpression of SOX9 significantly promoted the LX-2 cells migration (Fig. 5I, J).

### miR-139-5p regulates HSCs activation depending on β-catenin/SOX9/TGF-β1 signaling pathway

**Fig. 6:**
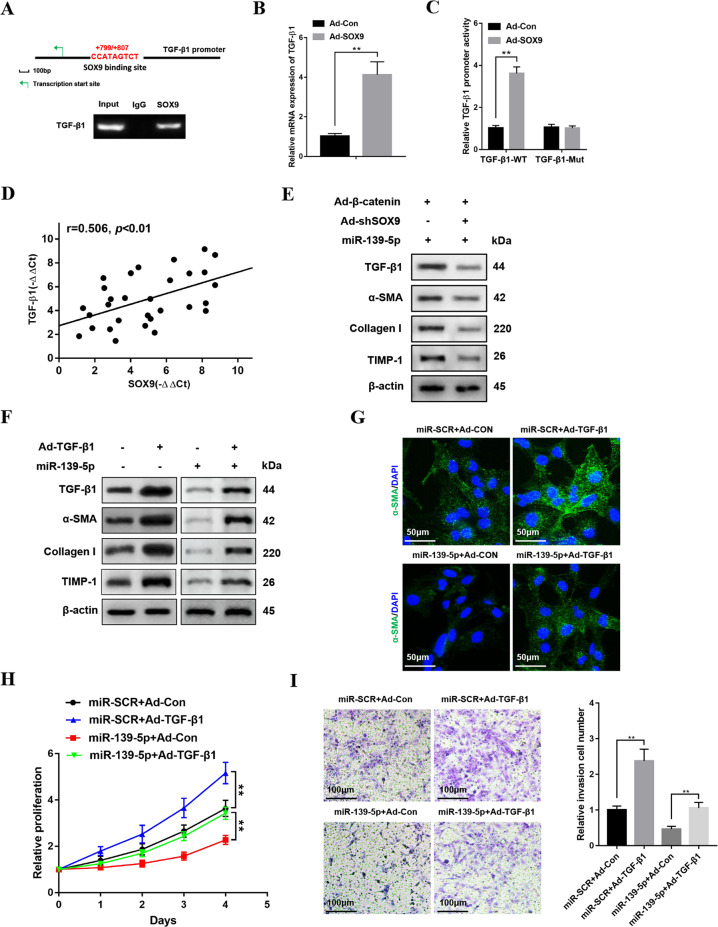
miR-139-5p regulates HSCs activation depending on β-catenin/SOX9/TGF-β1. **A** Schematic drawing of the putative site SOX9 binding with the human TGF-β1 promoter. ChIP assay of the LX-2 cells transfected with Ad-SOX9 were performed with anti-SOX9 or IgG antibody. Representative of three experiments. **B** The mRNA expression of TGF-β1 in activated LX-2 cells infected with Ad-SOX9 or Ad-Con. **C** The wild-type TGF-β1 promoter (TGF-β1-WT) or the mutant of TGF-β1 promoter (TGF-β1-MUT) luciferase reporter activity in activated LX-2 cells infected with Ad-SOX9 or Ad-Con was detected by dual-luciferase reporter assay. **D** Pearson’s correlation analysis was performed to analyze the correlation between SOX9 and TGF-β1 expression levels in patient fibrotic liver tissues, *n* = 30. **E** The protein levels of TGF-β1, α-SMA, Collagen-I, and TIMP-1 in LX-2-pre-miR-139-5p cells cells transfected with Ad-β-catenin and Ad-shSOX9 were examined by western blotting. Representative of three experiments. **F** The protein levels of TGF-β1, α-SMA, Collagen-I, and TIMP-1 in LX-2-pre-miR-139-5p cells infected with Ad-TGF-β1 were detected by western blotting. Representative of three experiments. **G** Immunofluorescence staining for α-SMA (green) in LX-2-pre-miR-139-5p cells transfected with Ad-TGF-β1 was observed by confocal laser microscopy. DAPI was used to stain the nuclei, blue. Original magnification×400, Representative of three experiments. **H** CCK8 assay was performed to detect the proliferation of LX-2-pre-miR-139-5p cells infected with Ad-TGF-β1. **I** The transwell assay of the LX-2-pre-miR-139-5p cells infected with Ad-TGF-β1 or Ad-Con was performed to analyze the migration capability, representative of three experiments. The number of cells was calculated from different fields. Graph represents mean ± SD. *P < 0.05, **P < 0.01.

To further investigate how SOX9 modulated HSCs activation and proliferation, bioinformatics analysis was utilized and we uncovered a putative homologous SOX9 binding sites within the human TGF-β1 promoter (+799/+807), which was further verified by ChIP assays (Fig. 6A). Accordingly, SOX9 overexpression increased the expression of TGF-β1 in HSCs (Fig. 6B). Then, the mutation of the sites that SOX9 bound with TGF-β1 promoter region abrogated the enhancement of TGF-β1 promoter activity triggered by SOX9 overexpression (Fig. 6C). In addition, the positive correlation between the levels of TGF-β1 and the expression of SOX9 was detected in patient fibrotic liver tissues (Fig. 6D). In addition, downregulation of SOX9 inhibited TGF-β1 expression and profibrotic markers in activated HSCs transfected with miR-139-5p mimics and Ad-β-catenin (Fig. 6E). To study the role of TGF-β1 in HSCs activation, TGF-β1 adenovirus was used to overexpress TGF-β1 in LX-2 cells transfected with miR-139-5p mimics. The overexpression of TGF-β1 could promote the expression of profibrotic markers including α-SMA, Collagen-I, and TIMP-1 in LX-2 cells transfected with miR-139-5p mimics (Fig. 6F), further verified by enhanced α-SMA immunofluorescence staining (Fig. 6G). Consistently, the overexpression of TGF-β1 significantly promoted LX-2 cells proliferation and migration (Fig. 6H, I). Collectively, these results demonstrates that miR-139-5p regulates HSCs activation depending on β-catenin/SOX9/TGF-β1 signaling pathway.

### Downregulation of lncRNA NEAT1 and overexpression of miR-139-5p alleviate liver fibrosis in mice

**Fig. 7:**
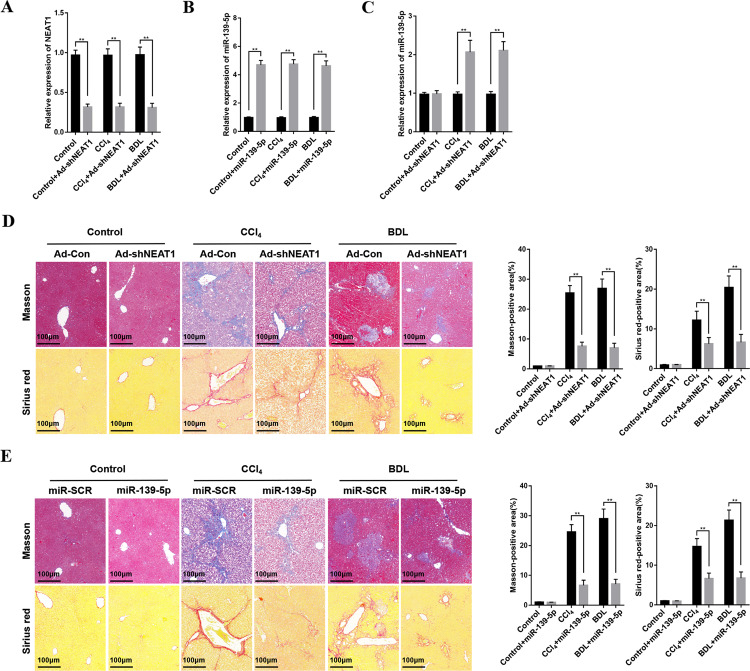
Downregulation of NEAT1 and overexpression of miR-139-5p alleviates hepatic fibrosis in mice. **A** The expression levels of NEAT1 was examined in liver tissues from fibrotic mice transfected with Ad-Con or Ad-shNEAT1. *n* = 6/group. **B** The expression levels of miR-139-5p in liver tissues from fibrotic mice transfected with agomir control and ago-miR-139-5p were examined by qRT-PCR. *n* = 6/group. **C** The expression levels of miR-139-5p in liver tissues from fibrotic mice transfected with Ad-Con or Ad-shNEAT1 were detected. *n* = 6/group. **D** The liver tissues from fibrotic mice transfected with Ad-Con or Ad-shNEAT1 were treated with Masson staining and Sirius red staining, *n* = 6/group, original magnification×200. **E** The liver tissues from fibrotic mice infected with agomir control and ago-miR-139-5p were treated with Masson staining and Sirius red staining, *n* = 6/group, original magnification×200. Graph represents mean ± SD. *P < 0.05, **P < 0.01.

To further explore the pathogenic roles of NEAT1 and miR-139-5p, we used in vivo murine fibrotic models to investigate the influences of their expressional alterations on liver fibrosis. We transfected murine fibrotic models with adenoviral vectors expressing shRNA against NEAT1 (Ad-shNEAT1) and miR-139-5p mimics to downregulate NEAT1 and upregulate miR-139-5p in vivo. The transfection efficiency of Ad-shNEAT1 and miR-139-5p in liver tissues was detected by qRT-PCR (Fig. 7A, B). We found that miR-139-5p expression was upregulated in murine fibrotic liver tissues transfected with Ad-shNEAT1 (Fig. 7C). Masson and Sirius Red staining demonstrated that the downregulation of NEAT1 and the overexpression of miR-139-5p attenuated liver fibrosis in vivo (Fig. 7D, E). Together, all these results indicate NEAT1 downregulation and miR-139-5p overexpression alleviate liver fibrosis in mice.

### Downregulation of lncRNA NEAT1 and overexpression of miR-139-5p suppressed the expression of profibrotic markers in murine fibrosis models

**Fig. 8:**
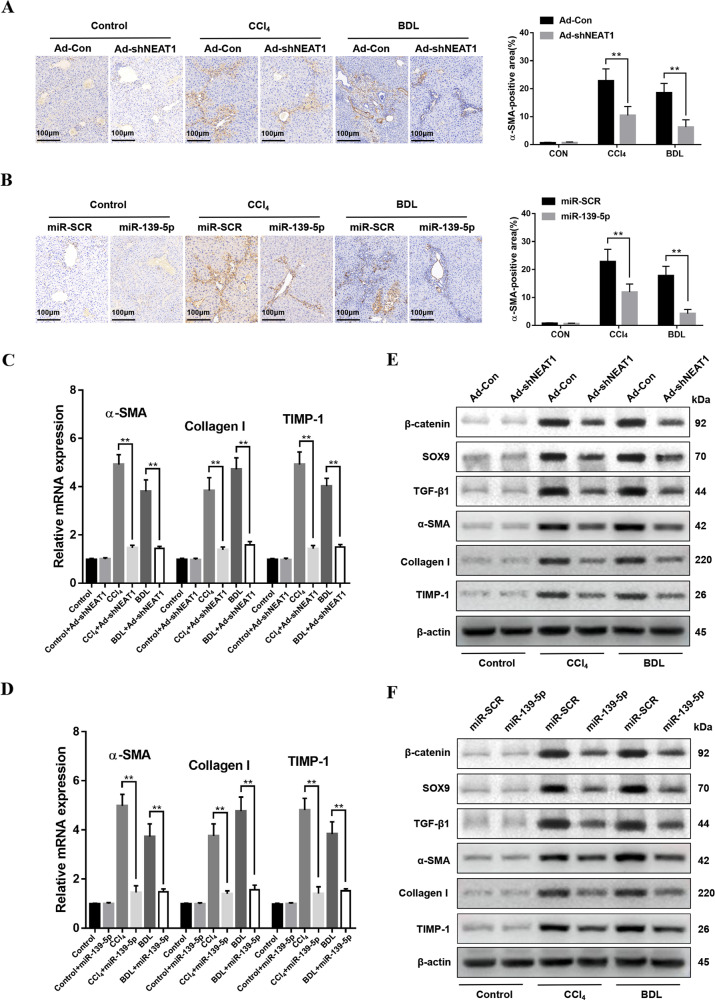
Downregulation of NEAT1 and overexpression of miR-139-5p suppressed the expression of profibrotic markers in murine fibrosis models. **A** The liver tissues from fibrotic mice injected with Ad-Con or Ad-shNEAT1 were treated with immunohistochemical staining, *n* = 6 mice for each group, original magnification × 200. **B** The liver tissues from fibrotic mice injected with agomir control or ago-miR-139-5p were treated with immunohistochemical staining, *n* = 6 mice for each group, original magnification × 200. **C** The mRNA expression levels of α-SMA, Collagen-I and TIMP-1 were detected in the liver tissues from the fibrotic mice injected with Ad-Con or Ad-shNEAT1 by qRT-PCR. *n* = 6/group. **D** The mRNA expression levels of α-SMA, Collagen-I and TIMP-1 were detected in liver tissues from the fibrotic mice injected with agomir control or ago-miR-139-5p by qRT-PCR. *n* = 6/group. **E** The protein levels of β-catenin, SOX9, TGF-β1, α-SMA, Collagen-I, and TIMP-1 in liver tissues from untreated mice and fibrotic mice transfected with Ad-Con or Ad-shNEAT1 were detected by western blotting. Representative of three experiments. **F** The protein levels of β-catenin, SOX9, TGF-β1, α-SMA, Collagen-I, and TIMP-1 in liver tissues from untreated mice and fibrotic mice transfected with the agomir control and ago-miR-139-5p were detected by western blotting. Representative of three experiments. Graph represents mean ± SD. *P < 0.05, **P < 0.01.

The immunohistochemistry of α-SMA indicated that NEAT1 downregulation and miR-139-5p overexpression suppressed the expression levels of α-SMA (Fig. 8A, B). qRT-PCR further indicated that NEAT1 downregulation and miR-139-5p overexpression suppressed the genes expression of α-SMA, Collagen-I, and TIMP-1 (Fig. 8C, D). The protein expression levels of profibrotic markers β-catenin, SOX9, TGF-β1, α-SMA, Collagen-I, and TIMP-1 in murine fibrotic liver tissues showed the same results (Fig. 8E, F). Moreover, we found that inhibition of miR-139-5p under the downregulation of lncRNA NEAT1 exacerbated liver fibrosis in vivo, which was validated by the mRNA levels of miR-139-5p (Fig. S1A), Masson staining and Sirius red staining (Fig. S1B, C), α-SMA immunohistochemical staining (Fig. S1D, E) in liver tissues from Ad-shNEAT1 transfected fibrotic mice treated with miR-SCR or miR-139-5p inhibitor. Meanwhile, the protein levels of β-catenin, SOX9, and TGF-β1 demonstrated that inhibition of miR-139-5p under the downregulation of lncRNA NEAT1 promoted the activation of β-catenin/SOX9/TGF-β1 pathway (Fig. S1F). The mRNA and protein levels of profibrotic markers α-SMA, Collagen-I, and TIMP-1 in murine fibrotic liver tissues further validated above results (Fig. S1G-I). Collectively, these results demonstrate that LncRNA NEAT1 exacerbated liver fibrosis by suppressing the expression of miR-139-5p. The downregulation of NEAT1 and overexpression of miR-139-5p suppress the expression of profibrotic markers in murine fibrosis models via targeting β-catenin/SOX9/TGF-β1 pathway (Fig. S2).

## Discussion

Liver fibrosis is caused by almost all chronic liver diseases, resulting in the progress of cirrhosis and related complications [29]. Recent studies have enriched our knowledge on liver fibrosis. However, the gap between basic research and clinical practice has not been dealed with due to lacking comprehension of the disease [30]. Hence, it is significant to explore the molecular mechanisms underlying liver fibrogenesis.

Our study explored the pathogenic roles of lncRNA NEAT1 and its interplay with the miR-139-5p in HSCs activation and the development of liver fibrosis. We uncovered for the first time that NEAT1 bound with miR-139-5p in activated HSCs to promote β-catenin expression, regulating HSCs proliferation and migration. Furthermore, our study proved that β-catenin played its fibrosis-promoting roles in activated HSCs by binding with SOX9 and positively regulating TGF-β1, which promoted liver fibrogenesis. The roles of NEAT1, and miR-139-5p on liver fibrosis were finally confirmed by in vivo murine fibrotic model. Herein, we uncovered a new NEAT1/miR-139-5p axis promoting HSCs activation and driving liver fibrosis development by modulating the β-catenin/SOX9/TGF-β1 signaling, providing potential targets for the early diagnosis and treatment of liver fibrosis.

LncRNAs, a group of long non-coding transcripts with a length of over 200 nucleotides, mainly act as sponges of target miRNAs and modulate gene expression post-transcriptionally^6^. Previous studies have shown that the networks between lncRNAs and miRNAs performed important regulatory roles in the pathogenesis of various human cancers and fibrotic diseases [31]. Among quantities of lncRNAs relevant to fibrogenesis, NEAT1 is a lncRNA originally serving as mammary development regulator, which was later verified to be intimately associated with fibrotic diseases [32]. Although NEAT1 was shown to regulate liver fibrosis and HSCs activation, the specific signaling pathways mediating its roles in liver fibrosis remain poorly known. Our study validated that the expression of NEAT1 was high in fibrotic liver and activated HSCs, negatively correlating with miR-139-5p expression. According to bioinformatics analysis, we predicted that NEAT1 could repress miR-139-5p expression, verified further by the dual-luciferase reporter assay and RIP methods in this study. Our study also demonstrated that the co-localization of NEAT1 and miR-139-5p were mainly in the cytosols of activated HSCs by FISH assay. Through regulating NEAT1 and miR-139-5p expression in HSCs, our study suggested that NEAT1 suppressed the expression of miR-139-5p and regulated HSCs activation. Our results promoted the non-coding RNA-mediated anti-liver-fibrosis development and provided novel insights into the molecular pathogenesis of liver fibrosis. Above all, our study showed that miR-139-5p could also be targeted by NEAT1, and their involvements in liver fibrogenesis deserved further investigations.

As mentioned above, increased β-catenin expression was intimately associated with HSCs activation and the progress of liver fibrosis [33]. Our study verified the significant increase of β-catenin expression in fibrotic liver tissues and HSCs. Moreover, our study indicated that the expression of β-catenin was positively relevant to NEAT1 but negatively relevant to the expression of miR-139-5p in fibrotic liver tissues. Nevertheless, the mechanisms responsible for markedly increased β-catenin expression in HSCs are little known. We used bioinformatics analysis and confidently confirmed that miR-139-5p could directly bind with the 3’UTR regions of β-catenin by the assay of dual-luciferase reporter, thus leading to the altered expression of β-catenin in HSCs. This is the direct evidence of molecular interplay between miR-139-5p and β-catenin. Above all, our study clearly indicated that miR-139-5p suppressed β-catenin expression and HSCs activation. Our study revealed for the first time that NEAT1 promoted HSCs activation through binding with miR-139-5p and suppressing miR-139-5p expression, further resulting in increased β-catenin expression in activated HSCs. In light of the divergent roles of β-catenin in other fibrotic diseases including renal [34], cardic [35], and pulmonary [36] fibrosis, further investigations of the NEAT1/miR-139-5p/β-catenin axis would promote full comprehension of epigenetic modulation of human fibrosis-related diseases.

Furthermore, the sex-determining region Y box (SOX) transcription factor family is reported to regulate a various biological behavioral process [37, 38]. SOX9 is a member of this group and taken for an important regulator of sex determination [39] and chondrocyte differentiation [40]. Previous study has found that SOX9 also advanced the progression of fibrotic diseases. For instance, a study demonstrated that SOX9 deletion in cardiomyocytes suppressed cardiac hypertrophy and fibrosis [41]. Another study suggested that SOX9 expression could be induced by canonical Wnt/β-catenin signaling in intestinal crypts [42]. For lung dieases, excessive activation of the Wnt pathway increased SOX9-expressing epithelial domain in the peripheral lung [43]. Activation of β-catenin increased the expression of SOX9 in peripheral epithelial progenitors, but deletion of β-catenin inhibited the expression of SOX9. All these studies confirmed that SOX9 could be directly or indirectly induced by the canonical Wnt/β-catenin pathway [41]. A recent study found that miR-145 could inhibited AKT/GSK-3β/β-catenin signaling pathway, which targeted SOX9 in fibroblasts, and alleviated cardiac fibrosis. However, the interplay between SOX9 amd β-Catenin in liver fibrosis remains poorly known. Our study indicated that β-catenin bound with SOX9 to promote HSCs activation and liver fibrosis. Moreover, previous studies demonstrated that TGF-β1 induced HSCs activation and promoted liver fibrosis [44, 45]. In this study, we found that β-catenin bound with SOX9 and promoted TGF-β1 transcription to activate HSCs and induce liver fibrosis according to bioinformatic analysis.

In summary, our study reported that the lncRNA NEAT1 targeted miR-139-5p to increase the expression of β-catenin in HSCs, which effectually promoted HSCs activation. Furthermore, the activation-promoting role of the NEAT1/miR-139-5p/β-catenin axis in HSCs was mediated by binding with SOX9 and increasing TGF-β1 expression, which further promoted HSCs activation. These findings unveiled a novel non-coding RNA-mediated liver fibrogenesis and laid the foundation for exploiting new anti-fibrosis treatments.

## Materials and methods

### Human liver specimens

Liver tissues (*n* = 30) were obtained from patients with liver fibrosis, who underwent liver resection in the First Affiliated Hospital of Nanjing Medical University. The control group (*n* = 30) were the para-hemangioma normal tissues from patients with hepatic hemangioma, who underwent surgical resection. Informed consent was obtained from all subjects. This study was endorsed by the Ethics Committee of the First Affiliated Hospital of Nanjing Medical University (number 2020-SR-511).

### Experimental animal models

Male C57BL/6 J mice used in this study were 8 weeks old and fed during the specific pathogen-free conditions with sterilized water and food. Hepatic fibrosis models were developed through carbon tetrachloride (CCl_4_) or bile duct ligation (BDL). Briefly, mice were intraperitoneally injected with CCl_4_ (10% in olive oil, 2 mL/kg, twice a week for 8 weeks) or vehicle (olive oil) to develop fibrosis and were sacrificed 48 h after the last injection. For BDL models, a common bile duct was exposed and ligated with 6-0 silk suture two times after midline laparotomy, abdomen was closed by a 6-0 suture, and mice were allowed to wake up on a heating pad. Controls were performed with a sham operation without ligating common bile duct. Mice were anesthetized with isoflurane and sacrificed at 2 weeks after BDL. The mouse liver tissues were collected for further study. All animal procedures were performed strictly following the recommendations in the protocol (number NMU08-092) endorsed by the Institutional Animal Care and Use Committee of Nanjing Medical University.

### HSCs cell culture

Human HSC line LX-2 cells were acquired from the Cell Center of Shanghai Institutes for Biological Sciences. Cells were cultured in DMEM containing 100 U/mL penicillin G sodium salt, 100 U/mL streptomycin sulfate, and 10% fetal bovine serum (Gibco, Carlsbad, CA, USA). LX-2 cells were treated with 10 ng/mL TGF-β1 for 24 h in DMEM without serum, respectively, for activation.

### miR-139-5p and virus transfections

miR-139-5p mimics were purchased (Gema, Shanghai, China). Adenoviruses (ViaGen, Shandong, China) expressing NEAT1, shRNA against NEAT1, β-catenin, SOX9, TGF-β1 and Vector (designated as Ad-NEAT1, Ad-shNEAT1, Ad-β-catenin, Ad-SOX9, Ad-TGF-β1 and Ad-Con) were used. Mice were injected with 20 nmol/200 µL agomir control (miR-SCR) or ago-miR-139-5p (Gema, Shanghai, China) via tail vein. Mice were injected with 20 nmol/200 µL antagomir control (miR-SCR) or antago-miR-139-5p (miR-139-5p inhibitor) (Gema, Shanghai, China) via tail vein. 1 × 10^9^ pfu/100 μL ad-shNEAT1 were injected into mice every two weeks from 2 weeks after CCl_4_ injections. Agomir control, antagomir control, ago-miR-139-5p or antago-miR-139-5p were injected into other mice twice a week from 2 weeks after CCl_4_ injections. Mice were sacrificed and liver tissues were collected at the end of the treatment. For BDL, Ad-shNEAT1 (1 × 10^9^ pfu/100 μL) were injected into mice via tail vein after BDL. Ago-miR-139-5p, antago-miR-139-5p, agomir control or antagomir control were injected into other mice twice a week via tail vein after BDL. After two weeks, mice were sacrificed and liver specimens were collected.

### Fluorescence in situ hybridization (FISH)

FISH method was performed to assess the expression of lncRNA NEAT1 and miR-139-5p in activated LX-2 cells with the FISH tag RNA multicolor kit (Thermo Fisher Scientific, Waltham, MA, USA) according to the manufacturer’s instructions. Specific FISH probes to NEAT1 and miR-139-5p were designed and synthesized by Servicebio (Wuhan, China). Dig-labeled NEAT1 probes and Cy3-labeled miR-139-5p probes were used in the hybridization. The signals of Dig-labeled probes were examined with a tyramideconjugated Alexa 488 fluorochrome TSA kit. The signals of Cy3-labeled miR-139-5p probes were determined with Cy3-Streptavidin (Life Technologies Corporation, Shanghai, China). Nuclei were counterstained with 4,6-diamidino-2-phenylindole dihydrochloride (DAPI). A confocal laser scanning microscope (Leica Microsystems, Mannheim, Germany) was used to analyze all images. The sequences of the FISH probe are shown as follows: NEAT1: 5′ CTCCCAGCGTTTAGCACAACACAATGACACC 3′; miR-139-5p: 5′ ACTGGAGACACGTGCACTGTAGA 3′.

### qRT-PCR

Total RNA was extracted from cells and liver tissues with a miRNeasy mini kit (QIAGEN, Valencia, CA, USA). Reverse transcription was performed with the Transcriptor First Strand cDNA Synthesis Kit (Roche, Indianapolis, IN, USA) according to the manufacturer’s instructions. Cytoplasmic or nuclear NEAT1 was extracted from HSCs with cytoplasmic and nuclear RNA purification kits (Norgen, Thorold, Canada). The levels of U6 and miR-139-5p was detected with the TaqMan miRNA assay system (Life Technologies Corporation, Shanghai, China). qRT-PCR was used to detect gene expression with SYBR green (Life Technologies Corporation, Shanghai, China). Results were normalized against β-actin expression and miR-139-5p against U6 snRNA, respectively. The expression levels were calculated by the 2^˗ΔΔCT^ method. The primers used for the amplification were as follow: human NEAT1, forward 5′-GUCUGUGUGGAAGGAGGAATT-3′, reverse 5′-UUCCUCCUUCCACACAGACTT-3′; mouse NEAT1, forward 5′-CTGGTTTATCCCAGCGTCAT-3′, reverse 5′-CTTACCAGACCGCTGACACA-3′; human miR-139-5, forward 5′-TGGAGACGCGGCCCTGTT-3′, reverse 5′-TCTACAGTGCACGTGTCT-3′; mouse miR-139-5p, forward 5′-TGGAGACGCGGCCCTGTT-3′, reverse 5′-TCTACAGTGCACGTGTCT-3′; human U6, forward 5′-CTCGCTTCGGCAGCACATA-3′, reverse 5′-AACGATTCACGAATTTGCGT-3′; mouse U6, forward 5′-CTCGCTTCGGCAGCACATA-3′, reverse 5′-AACGATTCACGAATTTGCGT-3′; human β-catenin, forward 5′-AGCTTCCAGACACGCTATCAT-3′, reverse 5′-CGGTACAACGAGCTGTTTCTAC-3′; human SOX9, forward 5′-AGCGAACGCACATCAAGAC-3′, reverse 5′-CTGTAGGCGATCTGTTGGGG-3′; human TGF-β1, forward 5′-CAATTCCTGGCGATACCTCAG-3′, reverse 5′-GCACAACTCCGGTGACATCAA-3′; human β-actin, forward 5′-CATGTACGTTGCTATCCAGGC-3′, reverse 5′-CTCCTTAATGTCACGCACGAT-3′; mouse α-SMA, forward 5′-GTCCCAGACATCAGGGAGTAA-3′, reverse 5′-TCGGATACTTCAGCGTCAGGA-3′; mouse Collagen I, forward 5′-GCTCCTCTTAGGGGCCACT-3′, reverse 5′-CCACGTCTCACCATTGGGG-3′; mouse TIMP-1, forward 5′-CGAGACCACCTTATACCAGCG-3′, reverse 5′-ATGACTGGGGTGTAGGCGTA-3′; mouse β-actin,forward 5′-GGCTGTATTCCCCTCCATCG-3′, reverse 5′-CCAGTTGGTAACAATGCCATGT-3′.

### Luciferase reporter assay

The wild-type or mutant NEAT1 was subcloned into pGL3 basic vector (Promega, Madison, USA). miR-139-5p mimics (RiboBio, Guangzhou, China) were cotransfected with 10 µg pLUC-WT-NEAT1 or pLUC-MUT-NEAT1 into HEK-293T cells. The wild-type or mutant TGF-β1 was subcloned into pGL3 basic vector (Promega, Madison, USA). miR-139-5p mimics (RiboBio, Guangzhou, China) were cotransfected with 10 µg pLUC-WT-TGF-β1 or pLUC-MUT-TGF-β1 into HEK-293T cells. Dual Luciferase Reporter Assay System (Promega, Madison, USA) was used to detect luciferase activity according to the manufacturer’s instruction.

### RIP assay

The EZ-Magna RIP kit (Millipore, MA, USA) was used to perform the RIP experiment according to the manufacturer’s instructions. RIP lysis buffer was used to lyse cells and magnetic beads conjugated to human anti-Ago2 antibody (Millipore, MA, USA). Isotype-matched IgG (Millipore, MA, USA) served as a negative control. Proteinase K was utilized to incubate samples, then immunoprecipitated RNA was isolated. NEAT1 level in the precipitates was detected using qRT-PCR.

### RNA pull-down assay

Biotin was used to mark purified RNAs with Pierce RNA 3’End Desthiobiotinylation Kit (Thermo Fisher Scientific, MA, USA). Briefly, LX-2 cells were transfected with Bio-miR-139-5p-Wt, Bio-miR-139-5p-Mut, or Bio-miR-SCR for 48 h, then the cells were washed with PBS and incubated in a lysis buffer for 10 min. To eliminate RNA and protein complexes, lysis buffer containing RNase-free BSA and yeast tRNA (Sigma, St.Louis, MO, USA) was used to block the beads. Streptavidin-coated magnetic beads (Life Technologies, CA, USA) were used to incubate the lysates at 25°C for 2 h and were washed twice with lysis buffer, three times with low-salt buffer, and once with high-salt buffer. TRIzol reagent (Life Technologies, CA, USA) was used to isolate the bound RNAs. The expression of NEAT1 was detected by qRT-PCR.

### Western blotting analysis

Proteins were extracted from liver tissues and cells with ice-cold lysis buffer (50 mM Tris, 0.1% sodium dodecyl sulfate, 300 mM Nacl, 1% Triton-100, 1% sodium deoxycholate). Proteins were treated with SDS-PAGE and transferred to polyvinylidene fluoride (PVDF) membranes (Millipore, MA, USA). The membranes were incubated with β-catenin, SOX9, α-SMA, Collagen I, TIMP-1, β-actin rabbit mAbs (Cell Signaling Technology, MA, USA), and TGF-β1 rabbit mAbs (Abcam, Cambridge, UK) overnight at 4°C after blocking, followed by secondary goat anti-rabbit IgG (Cell Signaling Technology, MA, USA) at 37°C for 1 h. β-actin served as an internal control.

### Histology, immunohistochemical and immunofluorescence analysis

Liver tissues were fixed in 4% buffered formalin. Liver tissue sections (4 µm thickness) were treated with the Masson and Sirius Red staining to determine the degree of collagen deposition. For immunohistochemical staining, primary antibodies of β-catenin, SOX9 or α-SMA (Cell Signaling Technology, MA, USA) were used to incubate liver tissue sections. HRP-Polymer-conjugated antibody served as the secondary antibody. Then, 3,3’-diaminobenzidine tetrachloride was used. The nuclei were counterstained with hematoxylin. The expression of β-catenin and SOX9 in LX-2 cells were determined by immunofluorescence with anti-mouse β-catenin mAb and anti-rabbit SOX9 mAb (Cell Signaling Technology, MA, USA). Next, LX-2 cells were incubated with secondary goat anti-mouse Texas Green-conjugated IgG or secondary goat anti-rabbit Texas Red-conjugated IgG (Sigma, St. Louis, MO, USA). DAPI was used to stain the nuclei. The slides were washed twice with PBS and observed with confocal microscopy (ZEISS, Oberkochen, Germany) in accordance with the manufacturer’s instructions. Positive cells were counted blindly in 10 HPF/section (×400).

### Immunoprecipitation analysis

The protein concentration of LX-2 cells was detected with BCA protein concentration assay kit (Thermo Fisher Scientific, MA, USA). Immunoprecipitation (IP) was performed with 1 mg total protein lysates from each sample. Then, the protein lysates were incubated with rabbit polyclonal IgG control antibody, rabbit monoclonal anti-β-catenin (Cell Signaling Technology, MA, USA) or mouse monoclonal anti-SOX9 (Santacruz Biotechnology, CA, USA). Next, the lysates were rotated at 4 °C for 4 h. Afterwards, the lysates were added with 25 μL protein A/G PLUS-Agarose and resuspended and the mixture rotated for another 2 h continuously. The eluted proteins were immunoblotted with the rabbit monoclonal anti-β-catenin (Cell Signaling Technology, MA, USA) or mouse monoclonal anti-SOX9 (Santacruz Biotechnology, CA, USA) after washing and denaturing with immunoprecipitation buffer.

### Chromatin immunoprecipitation assays

The chromatin immunoprecipitation (ChIP) assays were carried out with a ChIP assay kit (Cell Signaling Technology, MA, USA) in accordance with the manufacturer’s instructions. IgG and anti-SOX9 antibodies were used to immunoprecipitate the chromatin. The DNA was purified, and the qRT-PCR was performed to analyze the bound sequences. The primers were as follows: TGF-β1, forward: 5′ CACTCCTTTGTCTTGGAACTGTC 3′, reverse: 5′ GGCAACAAGGATCTCCCACT 3′.

### Flow cytometric (FCM)

Cell cycle experiments were performed with Cell Cycle Analysis Kit (Beyotime, Shanghai, China) in accordance with the manufacturer’s instructions. In brief, LX-2 cells were digested with trypsin. Next, LX-2 cells were centrifuged at 1000 rpm for 5 min. The cells were fixed with 70% ethanol prior to storage at −20°C overnight. Before FCM examination, cells were incubated with RNase (50 µg/mL). PI staining solution (500 µL) was used to stain cells for 30 min at room temperature to perform cell cycle analysis. Cells were stained with PI (Sigma, Saint Louis, MO, USA) and Annexin V-FITC (BD Biosciences, CA, USA) to perform apoptotic analysis in accordance with the manufacturer’s instructions.

### Cell proliferation and migration assays

For cell proliferation analysis, LX-2 cells were seeded into 96-well plates (3×10^3^cells per well). ATP activity were detected with Cell Counting Kit-8 (Dojindo, Kumamoto, Japan) at specific time points in accordance with the manufacturer’s instructions. For the cell migration assay, cells were seeded in the upper chamber of Transwell with serum-free DMEM (2×10^5^cells per well). The lower chamber was added with DMEM containing 10% FBS acting as chemoattractant. Cells migrating onto the lower surface of the filter were fixed and stained with crystal violet after incubation for 12 h. Meanwhile, for the detection of cell migration capability, a wound-healing assay was also performed following cells transfection. Briefly, 4×10^5^ cells per well were seeded and cultured into 24-well plates for 24 h. Afterwards, a sterile pipette tip was used to make a straight scratch in the middle of the cell monolayer, and wound-healing status was ultimately observed under microscopy.

### Statistical analysis

The data were shown as mean ± SD. Statistical analysis was performed with Student’s t-test or one-way analysis of variance on SPSS 20.0 software (Chicago, IL, USA). *P* < 0.05 (two-tailed) was considered to be statistically significant. Correlations between measured variables were tested by Spearman’s rank correlation analysis.

## Supplementary information

Supplemental Figure 1

Supplemental Figure 2

Supplemental Figure legends

Attribution of Authorship

## Data Availability

The data supporting the findings of this study are available from the corresponding author upon reasonable request.
