# Supplementary figures and images for: miR-139-5p sponged by LncRNA NEAT1 regulates liver fibrosis via targeting β-catenin/SOX9/TGF-β1 pathway
Source: Cell Death Discov. 2021 Sep 16;7:243. doi: 10.1038/s41420-021-00632-8 (PMC8446030; doi:10.1038/s41420-021-00632-8)

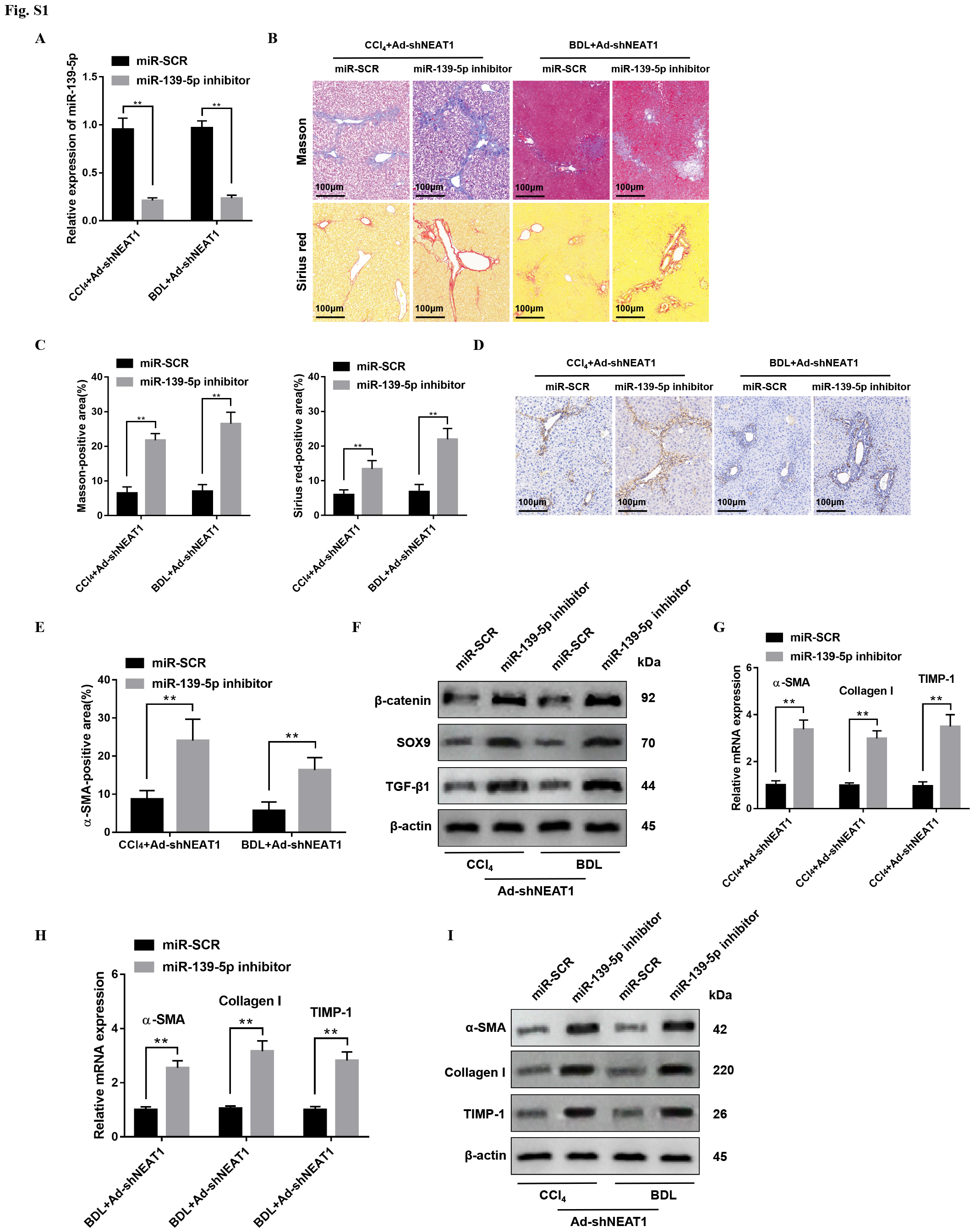

Supplement: Supplementary file 1 — Supplemental Figure 1 [file 41420_2021_632_MOESM1_ESM.tif]

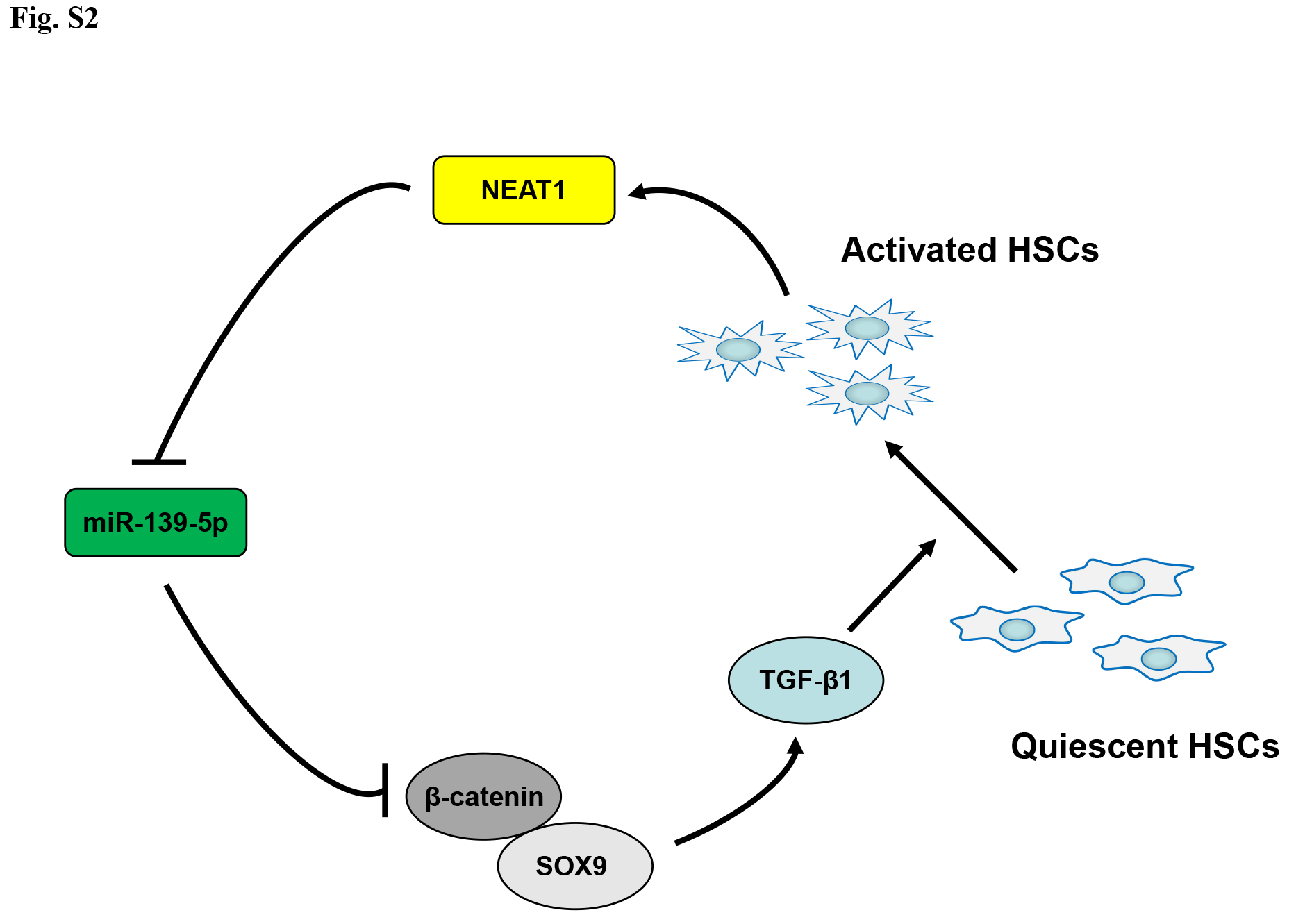

Supplement: Supplementary file 2 — Supplemental Figure 2 [file 41420_2021_632_MOESM2_ESM.tif]
