# Supplementary material for: miR-139-5p sponged by LncRNA NEAT1 regulates liver fibrosis via targeting β-catenin/SOX9/TGF-β1 pathway
Source: Cell Death Discov. 2021 Sep 16;7:243. doi: 10.1038/s41420-021-00632-8 (PMC8446030; doi:10.1038/s41420-021-00632-8)
Supplement: Supplementary file 3 — Supplemental Figure legends [file 41420_2021_632_MOESM3_ESM.docx]

**Supplemental Figure legends**

**Fig. S1 Inhibition of miR-139-5p under the downregulation of lncRNA NEAT1 exacerbated liver fibrosis *in vivo*.**

**A** The expression levels of miR-139-5p in liver tissues from Ad-shNEAT1 transfected fibrotic mice treated with miR-SCR or miR-139-5p inhibitor were examined by qRT-PCR. n=6/group. **B** The liver tissues from Ad-shNEAT1 transfected fibrotic mice treated with miR-SCR or miR-139-5p inhibitor were subjected to Masson staining and Sirius red staining, n=6/group, original magnification×200. **C** The quantitation of Masson staining and Sirius red staining. n=6/group. **D** The liver tissues from Ad-shNEAT1 transfected fibrotic mice treated with miR-SCR or miR-139-5p inhibitor were subjected to α-SMA immunohistochemical staining, n = 6 mice for each group, original magnification×200. **E** The quantitation of α-SMA immunohistochemical staining. n=6/group. **F** The protein levels of β-catenin, SOX9, and TGF-β1 in liver tissues from Ad-shNEAT1 transfected fibrotic mice treated with miR-SCR or miR-139-5p inhibitor were detected by western blotting. Representative of three experiments. **G** The mRNA expression levels of α-SMA, Collagen-I and TIMP-1 were detected in the liver tissues from Ad-shNEAT1 transfected CCl_4_-induced fibrotic mice treated with miR-SCR or miR-139-5p inhibitor by qRT-PCR. n = 6/group. **H** The mRNA expression levels of α-SMA, Collagen-I and TIMP-1 were detected in the liver tissues from the Ad-shNEAT1 transfected BDL-induced fibrotic mice treated with miR-SCR or miR-139-5p inhibitor by qRT-PCR. n = 6/group. **I** The protein levels of α-SMA, Collagen-I, and TIMP-1 in liver tissues from the Ad-shNEAT1 transfected fibrotic mice treated with miR-SCR or miR-139-5p inhibitor were detected by western blotting. Representative of three experiments. Graph represents mean±SD. ***P* < 0.01.

**Fig. S2 Schematic diagram showing the mechanisms of lncRNA NEAT1 and miR-139-5p in regulating liver fibrosis progression.**

LncRNA NEAT1 could sponge miR-139-5p and promote hepatic stellate cells (HSCs) activation by directly inhibiting the expression of miR-139-5p. miR-139-5p upregulation could suppress the expression of β-catenin. β-catenin could interact with SOX9 promoted HSCs activation. SOX9 could bind with the TGF-β1 promoter and promoted the transcription activity of TGF-β1. The upregulation of TGF-β1 further promoted HSCs activation. In conclusion, our study suggested that miR-139-5p sponged by lncRNA NEAT1 regulated liver fibrosis via targeting β-catenin/SOX9/TGF-β1 Pathway.
