# Supplementary material for: miR-139-5p sponged by LncRNA NEAT1 regulates liver fibrosis via targeting β-catenin/SOX9/TGF-β1 pathway
Source: Cell Death Discov. 2021 Sep 16;7:243. doi: 10.1038/s41420-021-00632-8 (PMC8446030; doi:10.1038/s41420-021-00632-8)
Supplement: Supplementary file 4 — Attribution of Authorship [file 41420_2021_632_MOESM4_ESM.pdf]

DECLARATION OF CONTRIBUTIONS TO ARTICLE

ADMC

Manuscript Number:	Journal Name:	
CDDISCOVERY-21-1600	*Cell Death Discovery*	(the 'Journal')

Proposed Title of the Contribution:

miR-139-5p Sponged by LncRNA NEAT1 Regulates Liver Fibrosis via Targeting β-catenin/SOX9/TGF-β1 Pathway (the 'Contribution)

Author(s):

Qi Wang, Song Wei, Qingfa Bu, Lei Li, Haoming Zhou, Wantong Su, Zheng Liu, Mingming Wang, Ling Lu (the 'Authors')

For all *CDDis*covery articles, each person named as an author in the published version must be able to show he or she has contributed substantially to the article.

Authorship credit should be based on 1) substantial contributions to conception and design, acquisition of data, or analysis and interpretation of data; 2) drafting the article or revising it critically for important intellectual content; and 3) final approval of the version to be published. Authors should meet conditions 1, 2 and 3.

Any person who cannot be shown to have made a substantial contribution to the article cannot be listed as an author in the final version. The name of any person who is deemed to have made a minor contribution can, however, appear in the Acknowledgments section of the article.

Please complete the table below to indicate the contributions of all named authors to the manuscript.

Author Full Name:	Specification of Contribution to the Manuscript:
Qi Wang	performed the experiments, analyzed the data and wrote the first draft of manuscript
Song Wei	performed the experiments, analyzed the data and participated critical discussion
Lei Li	performed the experiments, analyzed the data and participated critical discussion
Qingfa Bu	performed the experiments, analyzed the data and participated critical discussion
Haoming Zhou	performed the experiments and participated in data analysis and critical discussion
Wantong Su	performed the experiments and participated in data analysis and critical discussion
Zheng Liu	performed the experiments and participated in data analysis and critical discussion
Mingming Wang	performed the experiments and participated in data analysis and critical discussion
Ling Lu	contributed to the study concept, research design, and finalized the manuscript

Please complete the table below to indicate the contributions of all named authors to the figures.

Figure 1:

Qi Wang finished the qRT-PCR and prepared panel A-C, Qi Wang and Song Wei finished tissues staining, labelled the image and assembled the panel D. finished the qRT-PCR and prepared panel E-G.

Figure 2:

Lei Li performed generated the data and prepared panel A-D, Qi Wang finished cell staining and prepared panel E. Qingfa Bu generated the data and prepared panel F-H, Haoming Zhou generated the data and prepared panel I-K.

Figure 3:

Qi Wang and Song Wei labelled the image and assembled the panels A-C. Lei Li assembled the panels D and E. Qingfa Bu finished α-SMA staining, generated the data and prepared panel F. Haoming Zhou labelled the image and assembled the panels G-I.

Figure 4:

Qi Wang, Wangtong Su and Zheng Liu prepared panels A-C. Qi Wang and Mingming Wang labelled the image and assembled the panels D-G.

Figure 5:

Qi Wang and Song Wei labelled the image and assembled the panels A-E. Lei Li generated the data and prepared panel F-H. Qingfa Bu labelled the image and assembled the panels i-j.

Figure 6:

Haoming Zhou labelled the image and assembled the panels A-D. Wangtong Su finished the western blot, and assembled the panels E-F. Qi Wang performed Immunofluorescence staining and assembled panel G. Zheng Liu generated the data and prepared panels H-I.

Signed for and on behalf of the Author(s):	Print Name:	Date:
Qi Wang	Qi Wang	2021/03/31
